# Clinical Aspects of Stevens-Johnson Syndrome and Toxic Epidermal Necrolysis With Severe Ocular Complications in Brazil

**DOI:** 10.3389/fmed.2021.649369

**Published:** 2021-06-18

**Authors:** Tais Hitomi Wakamatsu, Myrna Serapião dos Santos, Telma Pereira Barreiro, Ana Estela Besteti Pires Ponce Sant'Anna, Fabíola Murta, Alexandre Xavier da Costa, Leonardo Guedes C. Marculino, Rafael Jorge Alves de Alcântara, Charles Costa de Farias, José Álvaro Pereira Gomes

**Affiliations:** ^1^Department of Ophthalmology, Federal University of São Paulo, São Paulo, Brazil; ^2^Moorfields Eye Hospital, London, United Kingdom

**Keywords:** Stevens-Johnson syndrome, toxic epidermal necrolysis, Brazil, clinical aspects, treatment, genetic predisposition

## Abstract

Stevens-Johnson syndrome (SJS) and toxic epidermal necrolysis (TEN) are acute and potentially fatal inflammatory vesiculobullous reactions that affect the skin and mucous membranes, and which are most often triggered by particular medications and infections. In Brazil, the drugs most frequently associated with TEN and SJS include cold medicine such as dipyrone and NSAIDs, followed by carbamazepine, phenobarbital, penicillin, and allopurinol. Genetic variations have been found to increase the risk of SJS/TEN in response to triggering factors such as medications. The most closely associated genes found in Brazilian cold-medicine-related SJS/TEN patients with severe ocular complications are HLA-A^*^66:01 in those of mixed African and European ancestry and HLA-B^*^44:03 and HLA-C^*^12:03 in those of solely European ancestry. Our classification system for grading ocular surface complication severity in SJS/TEN patients revealed the most severe complications to be limbal stem cell deficiency and dry eye. Changes to the conjunctival flora have also been observed in SJS/TEN patients. Our group identified bacterial colonization in 95% of the eyes (55.5% of which were gram-positive cocci, 25.5% of which were gram-negative bacilli, and 19% of which were gram-positive bacilli). Several new treatment options in the acute and chronic ocular management of the SJS/TEN patients have been described. This article highlights some Brazilian institutions' contributions to ocular surface care in both the acute phase (including the use of amniotic membrane transplantation) and the chronic phase (such as eyelid margin and fornix reconstruction, minor salivary gland transplantation, amniotic membrane and limbal transplantation, scleral contact lenses, anti-angiogenic eyedrops for corneal neovascularization, *ex-vivo* cultivated limbal epithelium transplantation, conjunctival-limbal autografting, oral mucosa transplantation, and keratoprosthesis).

## Introduction

Stevens-Johnson syndrome (SJS) and toxic epidermal necrolysis (TEN) are rare but life-threatening hypersensitivity reactions characterized by inflammatory vesiculobullous reaction of the skin and mucous membrane (1, 2). They are considered two of the most devastating ocular surface diseases in that they cause corneal damage and threaten vision. SJS and TEN are estimated to affect two to ten million people each year (3, 4). The mortality rate of SJS is estimated as 16.7% in Brazil (5). Several aspects of SJS and TEN are considered to be unmet needs in terms of management in different regions of the globe. In this review, we will highlight some clinical aspects of these diseases in Brazilian patients.

## Clinical Aspects of Brazilian Patients

### Epidemiology

In Brazil, ocular complications are reported to be the most common complication related to SJS/TEN (5). We retrospectively reviewed a total of 108 patients (61 females and 47 males) with SJS and TEN who were treated in the Ophthalmology Department of Paulista School of Medicine. Mean age at onset was 22.81 years of age (range of 7 months−58 years). Acute ocular manifestations consisted of corneal erosions and conjunctivitis. The prevalence of severe ocular complications (SOCs) varied, with the most common chronic presentation represented by dry eye syndrome, limbal stem cell deficiency (LSCD) and trichiasis (6).

### Etiology

SJS and TEN are commonly associated with pharmaceuticals and infectious agents. Of the 108 patients with SJS and/or TEN included herein, cold symptoms were reported by 73 patients (67.59%), and symptom onset was reported after the use of implicated medications in 98 patients (90.74%). The medications mostly commonly taken by the patients prior to symptom onset were dipyrone (41 patients; 37.96%), penicillin (13 patients; 12.04%), phenobarbital (9 patients; 8.33%), sulphonamides (8 patients; 7.41%), phenytoin (seven patients; 6.48%), and carbamazepine (six patients; 5.56%), followed by aspirin (four patients; 3.70%), allopurinol (three patients; 2.78%), amoxicillin (two patients; 1.85%), paracetamol (two patients; 1.85%), and lamotrigine (two patients; 1.85%). Cephalexin, ciprofloxacin, ibuprofen, ofloxacin, piroxicam, ipilimumab, zidovudine, and theophylline were each recorded in one patient (0.93% each). Finally, eight patients (7.41%) had taken an unknown pharmaceutical prior to symptom onset.

Human leukocyte antigens (HLAs) are highly polymorphic proteins that start the immunity process by introducing pathogen-derived peptides into T-cells (7). HLA typing in large patients samples involving different autoimmune diseases has revealed the occurrence of HLA alleles at higher frequencies in patients with certain diseases than in the general population; these studies have also demonstrated that carries of a specific HLA allele experience an increased risk of developing SJS and/or TEN. In a study of Japanese patients, Ueta et al. (8, 9) found that HLA-A^*^02:06 is closely associated with and HLA-A^*^11:01 is inversely associated with cases of SJS and TEN with SOCs. These same authors later found close to 80% of the reactions to be associated with cold medicine (CM) (10). In another study, they found HLA-A^*^02:06 and HLA-B^*^44:03 to be closely and independently associated with CM-related SJS and TEN (CM-SJS/TEN) with severe mucosal involvement, including SOCs (8). The association between CM-SJS/TEN and theses alleles was confirmed in a study that organized results by ethnicity in the consideration of Indian, Brazilian, and Korean populations (9). These findings support genetic predispositions for CM-SJS/TEN with SOCs. CM represents a group of medications marketed to help relieve symptoms of common cold. They include analgesics and antipyretics (such as dipyrone and acetaminophen) and non-steroidal anti-inflammatory drugs (NSAIDs), such as salicylates, propionic acid, acetic acid, enolic acid, anthranilic acid derivatives, selective cyclooxygenase-2 inhibitors, and sulphonamides).

We conducted a case-control study to determine any associations between HLA class I genes and CM-SJS/TEN with SOCs. Thirty-nine Brazilian patients with CM-SJS/TEN, 74 patients with SJS/TEN with SOCs, and 133 healthy Brazilian volunteers were enrolled. We found different alleles to be associated with CM-SJS/TEN with SOCs in Brazilians of mixed African and European and of solely European ancestry. The HLA-A^*^66:01 allele may be a marker for CM-SJS/TEN with SOCs in individuals of mixed African (OR, 12.2; 95% CI, 1.19–125.0; *P* = 0.03) and European ancestry (OR, 21.2; 95% CI, 0.97–465.0; *P* = 0.04), while HLA-B^*^44:03 (OR, 5.50; 95% CI, 1.47–20.50; *P* = 0.01) and HLA-C^*^12:03 (OR, 8.79; 95% CI, 1.83–42.20; *P* = 0.008) were found to be likely markers in those of solely European ancestry. We suggest that HLA-A^*^11:01 is a universal marker of resistance to CM-SJS/ TEN with SOCs (11).

In a recent study, we determined the association of HLA class I and II with dipyrone-related SJS/TEN with severe ocular complications in a Brazilian population. We found that HLA-B^*^44:03 (carrier frequency: *p* = 0.002, Pc = 0.02, OR = 8.8; gene frequency: *p* = 0.001, Pc = 0.01, OR = 7.5) and HLA-DQB1^*^04:02 (carrier frequency: *p* = 0.01, Pc = 1.08, OR = 11.4; gene frequency: *p* = 0.003, Pc = 0.03, OR = 12.6) were significantly associated with cases of dipyrone-related SJS/TEN with SOCs in the Brazilian population of European ancestry. HLA-C^*^05:01 (carrier frequency: *p* = 0.001, Pc = 0.01, OR = 19.4; gene frequency: *p* = 0.002, Pc = 0.02, OR = 15.0) was significantly associated with cases of dipyrone-related SJS/TEN with SOCs in the Brazilian population of mixed raced ancestry (12).

### Classification

A classification system provides a quantitative tool to compare outcomes in SJS/TEN. It is important to identify disease progression, documenting patient follow up, and helps to monitor the response to a specific treatment.

Sotozono et al. graded the severity of corneal, conjunctival and eyelid complications totalizing 13 components on a scale from 0 to 3. They showed that severity of chronic ocular manifestations in SJS/TEN is significantly correlated with the final visual outcome (13). Sharma et al. scored 12 ocular surface parameters ranging from 0 to 5 and demonstrated that correlated significantly with corrected distance visual acuity (14). Ong et al. analyzed 12 components of the ocular surface divided in three functional categories (inflammation, scarring and ocular morbidity) and validated this tool of clinical assessment for cicatrizing conjunctivitis (15).

To assess and grade the extent and severity of ocular surface manifestations of SJS, we proposed a grading system for assessing ocular surface manifestations. Ocular surface manifestations were separated into corneal complications, conjunctival complications, eyelid-related complications, and the presence or absence of dry eye disease. Nine components were graded on a scale from 0 to 3 based on their severity. This grading system also separated the complications into those of the cornea (epitheliopathy, opacity and LSCD), conjunctiva (inflammation and wound healing), and eyelid (keratinization and eye lash abnormalities), as well as dry eye status (Schirmer's test and corneal wound healing).

### Microbiome

Microbial communities and their genes—understood together as the microbiome—are spread throughout different areas of the human body. They maintain homeostasis and aid immunity against disease (16). The microbiome is affected by interactions with both environmental antigens and drugs, including antimicrobials. The bacterial genome of some mucosal surfaces alone is more than 100 times the size of the human genome (17). Other mucosal sites, such as the ocular surface, are paucibacterial, with less than a single bacterium per human cell (18, 19).

There seems to be an immunological relationship between the gut and other organs such as the eye. Gut microbiome abnormalities have been linked to a variety of ocular conditions including dry eye, diabetic retinopathy, glaucoma, macular degeneration, and infectious keratitis (20, 21). In an experimental model of Sjögren's syndrome, a dysbiotic intestinal microbiome was associated with worse presentation of ocular mucosal disease (21).

The microbiome in cases of SJS has also been investigated. Two studies found that the frequency of conjunctival culture positivity was much higher than that which was reported in healthy patients (22, 23). Coagulase-negative *staphylococci* and *Corynebacterium spp*. were the most commonly found species (22, 23). Half of the patients had multiple bacterial species in their flora, including pathogenic bacteria such as *Enterobacter spp, Serratia nonliquefaciens, Escherichia coli, Morganella morganii, Proteus mirabilis*, and *Haemophilus spp* (22). A more recent study compared the microbiomes from SJS patients to those of healthy subjects using conventional cultures and sequencing methods (24). Positive-cultured specimens were found in 60% of the SJS patients and in only 10% of the healthy subjects (24). *Corynebacterium* and *Streptococcus* were the bacteria genera detected most frequently. rRNA sequencing confirmed a wider diversity of microbial species and a greater proportion of pathogenic microorganisms in the eyes of SJS patients; the genera detected included *Pseudomonas, Staphylococcus, Streptococcus*, and *Acinetobacter* (24).

This altered microbiome found in SJS cases is likely associated with severe ocular surface abnormalities, including chronic epitheliopathy and reduction of the mucin layer of the tear film (24). In addition, it has been reported that SJS produces an abnormal innate immune response that may affect the balance between mucosal immunity and microorganism pathogenicity, inducing chronic-recurrent ocular surface inflammation (25). These changes can predispose patients to severe ocular infections that might impact the results of surgical procedures for ocular surface reconstruction, thus jeopardizing visual rehabilitation of these patients (26). In their prevention and control of infection in SJS patients, physicians are advised to start these patients on appropriate antibiotics prior to relevant procedures (24–26).

### Acute Phase Treatment

In the acute phase of SJS, patients exhibit a critical state of involvement of multiple organs and thus require the support of a multidisciplinary team. Ocular treatment in the acute phase consists of maintaining eye hygiene, intensive lubrication, the mechanical removal of membranes, prophylactic topical antibiotics, corticosteroids, and therapeutic contact lenses.

Amniotic membrane transplantation (AMT) to treat acute SJS is an option for severe cases. It can aid in epithelization, in addition to decreasing inflammation and the consequences thereof (entropion, symblepharon, and dry eye) (27, 28). If the procedure is not performed within 10 days of the beginning of this condition, severe vision-threatening complications can occur. In these cases, however, LSCD is inevitable and will require limbal stem cell transplantation (LSCT) or other surgeries used for ocular surface reconstruction (29). In AMT, the amniotic membrane covers the entire bulbar surface up to the fornices. Nylon sutures (10-0) can be used to secure the edge of the membrane to the lid margin, and the larger silk sutures can be passed through the eyelid as a mattress stitch to secure the membrane reflected into the fornix; fibrin glue can also be used (29).

### Chronic Phase Treatment

Ocular treatment during the chronic phase of SJS is considered one of the most substantial challenges in ophthalmology. The goals of treatment include restoration of eyelid function, dry eye management, and restoration of the ocular surface.

#### Eyelid Complications

##### Eyelid Margin Reconstruction

Mucous membrane transplants have been used for the reconstruction of fornices damaged by symblepharon formation, a common condition in chronic SJS patients (30). Nevertheless, some keratin accumulates on the eyelid margin, causing inflammation and irritation of the ocular surface.

Iyer et al. (31) reported on the use of the oral mucosa to reconstruct the eyelid margin by removing the keratinized area and grafting a mucous membrane from the lip using fibrin glue. They studied 54 eyes of 31 patients, with an improvement in 92.6% of patients (31). In our department, we prefer to use this technique by removing the lip mucosa with a mucotome to produce a thinner graft more similar to the conjunctiva and to suture it with 8-0 polyglactin suture.

#### Adnexal complications

##### Minor Salivary Gland Transplantation

Severe dry eye is known to be one of the main sequelae experienced by patients with SJS. The minor salivary glands located in the submucosa of the oral cavity can be used in the visual rehabilitation of patients with SJS. These glands are classified as labial, buccal, glossopalatine, and palatine. The minor labial salivary glands, which are present on the inner surface of the upper and lower lips, are the glands most commonly used in severe dry eye treatment.

Several authors have successfully described the transplantation of minor salivary glands for the treatment of severe dry eye in patients with SJS and severe eye burns (32–34). A thin labial mucosal graft removed with a mucotome (35) can be used to correct the symblepharon in association with the minor salivary gland transplantation, providing the eye not only with labial mucosa to serve as a lining, thus reestablishing the ocular surface, but also with the minor salivary glands which increase the amount of tear film (36).

In 2012, Sant'Anna et al. reported on 19 patients with SJS and severe symblepharon treated simultaneously with symblepharon correction through labial mucosal grafting obtained using a mucotome and autologous transplantation of minor salivary glands attached to the submucosa (36) and not full thickness mucosa as previously described by Murube et al. (37). The glandular tissue attached to the submucosa was implanted nasally in the lower and upper sacs. The results were satisfactory, and Schirmer's test results improved in all patients. Results were superior in patients in whom more than 10 glands were implanted. Therefore, this procedure managed to simultaneously treat severe symblepharon and implant minor salivary glands (36). Wakamatsu et al. also demonstrated the viability of minor salivary glands transplanted into the fornices of patients with dry eye by performing immunohistochemistry on graft biopsies with antibodies against lactoferrin, lysozyme, MUC1, and MUC16. The salivary gland units were found to be functional, with local production of proteins, enzymes, and mucins (38).

Vazirani et al. reported the transplantation of a complex consisting of mucosa, minor salivary glands, and muscle that removed and implanted en bloc in 19 patients (21 eyes) with cicatricial conjunctivitis. During the surgeries, the minor salivary glands were attached to the upper bulbar surface and anchored to the superior rectus muscle. Patients' visual acuity and Schirmer's test results improved (39).

#### Corneal complications

##### Anti-VEGF Treatment for Corneal Neovascularization

Corneal neovascularization is a common consequence of ocular manifestations and is associated with significant visual morbidity. Neovascularization triggers tissue scarring, stromal hemorrhage, lipid deposition, and corneal edema, which all have severely negative effects on visual acuity. Several factors, including vascular endothelial growth factor (VEGF) and platelet-derived growth factor (PDGF), regulate angiogenesis. Recent findings suggest that VEGF and PDGF inhibition may be effective treatment options for corneal neovascularization (40).

Our team performed a prospective, randomized, double blind, placebo-controlled study to evaluate the safety, efficacy, and stability of topical bevacizumab and sunitinib in the treatment of corneal neovascularization in patients with SJS (manuscript in preparation).

##### Scleral Contact Lenses

In recent years, several studies have considered the use of different scleral contact lenses (SCLs) to treat dry eye disease (DED). SCLs are typically indicated for severe DED in cases when conventional treatment fails. SCLs protect the cornea and conjunctivae because the covering they provide controls evaporation and maintains direct contact between fluid and the corneal epithelium. SCLs also protect the cornea from the mechanical trauma and abrasions that commonly result from irregular eyelid scarring and misdirected eyelashes that SJS patients often exhibit (41).

Weber et al. evaluated the efficacy of SCL treatment and how this treatment affects clinical tests used to determine severe SJS with DED. The SCL treatment positively impacted SJS patients' tear osmolarity and vital staining scores; it also improved their visual acuity, DED symptoms, and overall quality of life (42, 43).

##### Limbal Stem Cell and Amniotic Membrane Transplantation

Conjunctival-limbal grafting combined with AMT is a surgical procedure currently available for reconstruction of the ocular surface in cases of total LSCD (26, 44–48).

In 2003, Gomes et al. studied 10 eyes of 10 patients with LSCD secondary to SJS who were treated with AMT and a living-related conjunctival-limbal allograft. As we noted in our previous study, which relied on a follow-up period that averaged 16.7 months, “satisfactory ocular surface reconstruction was obtained in 2 eyes (20%), with reduced inflammation and vascularization and a mean epithelialization time of 3 weeks. Surgical failure was observed in four cases (40%), and complications (infection) occurred in four cases (40%). Visual acuity improved in four eyes (40%), remained stable in five eyes (50%), and decreased in one eye (10%)” (26).

In 2005, Santos et al. prospectively evaluated the survival of conjunctival-limbal grafts associated with AMT for LSCD and assessed the role of different effects and symptoms associated with LSCD, eyelid abnormalities, keratinization, dry eye, systemic immunosuppression, HLA compatibility, and keratoplasty (PKP) on surgical outcomes in a prospective, non-comparative, interventional study (47). Of the 31 patients with total LSCD who received conjunctival-limbal grafts and AMT, 11 of the cases (33%) were secondary to SJS. Ten eyes (30%) received a conjunctival-limbal autograft, and 23 (70%) received a conjunctival-limbal allograft from living HLA-matched donors; these counts represented all of the SJS patients included in the study. Grafts survived in 13 eyes (40%) at 1 year and in 11 eyes (33.3%) at 2 years; the cumulative graft survival rate was 33% at a mean follow-up time of 33 months. Among the SJS patients, the graft survival rate decreased significantly over time, with a survival rate of only 10% after 1 year (47).

Other researchers have found this type of procedure to have a substantial effect on graft survival in patients with SJS, dry eye, eyelid abnormalities, keratinization, and/or allogeneic conjunctival-limbal transplantation, regardless of HLA compatibility (*p* < 0.05). The researchers found preoperative dry eye to be the parameter that most highly predicted surgical outcome (*p* < 0.001) (47).

##### Corneal Transplantation

In aggressive and prolonged ocular surface morbidities, such as those caused by SJS/TEN, keratoplasty is employed largely to attempt to preserve of the ocular globe (49). Improvement in final visual function by corneal transplantation is a challenge in SJS patients. The prognosis is typically poor (50) due to the patients' high risk of immune rejection, persistent epithelial defection, infection, graft melting, and corneal perforation (22). Today, keratoplasty is considered for visual rehabilitation only after ocular surface restoration is completed (41, 51). Moreover, almost all SJS patients require systemic immunosuppression after corneal transplantation (26, 52).

In our department, we follow the same protocols described previously for keratoplasty in patients with acute and chronic SJS/TEN.

##### Keratoprosthesis

Patients with neovascularization, severe corneal opacity, and LSCD may have part of their vision restored for varying lengths of time through keratoprosthesis. The risks of operative and postoperative complications are greater in SJS/TEN patients than in other groups who undergo keratoprosthesis, and rates of both anatomical success (retention of the prosthesis) and functional success (improved visual acuity) are lower than among patients with other types of diseases. Corneal necrosis, infectious keratitis, microbial endophthalmitis, and glaucoma are some of the complications experienced by SJS/TEN patients at rates substantially higher than among patients with other conditions (53). Oliveira et al. reported the experience of our department in performing Boston Kpro type I. They evaluated and compared the results in subgroups distinguished by previous diagnosis (SJS/TEN, chemical burn and multiple graft failure). There was a tendence of lower retention rate in the SJS/TEN group and no statistically differences in visual acuity outcomes among the three groups was found (54).

Type I and II Boston keratoprosthesis devices and modified osteo-odonto keratoprosthesis (MOOKP) are the treatment options currently available. Boston Kpro type I may be indicated for some patients with relatively healthy ocular surfaces and eyelids, while Boston Kpro type II and MOOKP are reserved for cases with major abnormalities of the eyelid, keratinization of the conjunctiva, and/or severe dry eye. More recently we started to perform salivary gland transplantation before the implantation of Boston type I keratoprosthesis in a few selected SJS patients presenting severe dry eye with encouraging results. The decision between the latter two options depends on the surgical conditions available, the surgeon's experience, and local regulations (41).

Artificial corneal implants in SJS/TEN patients should be seen as a surgery of last resort and should be preceded by other visual rehabilitation methods.

#### Systemic Immunosuppression

The importance of continued immunosuppression for graft survival after ocular surface stem cell transplantation has been discussed previously. The systemic immunosuppression protocols used by our team to prevent rejection after allogenic LSCT is based on the combination of 1 mg/kg prednisone and 3 to 5 mg/kg cyclosporine administered orally and daily and, more recently, 1 mg/kg oral prednisone once daily, 4mg tacrolimus twice daily, and 1 g mycophenolate mofetil (MMF) twice daily. Oral prednisone is tapered progressively and discontinued after 8 weeks. Cyclosporine, tacrolimus, and MMF dosages are tapered after 8 to 12 weeks but are administered indefinitely. Blood cell counts, kidney function, and liver function, are tested monthly (47, 55–57).

## Discussion and Conclusions

SJS and TEN commonly involve ocular complications. The acute conjunctival inflammation seen in SJS and TEN patients leads to chronic scarring of the ocular surface, often the most devastating long-term sequela in these patients. The sequelae experienced required that the initial treatments being instituted within windows of opportunity. Prevention of complications is the most reliable way to minimize vision loss. Despite improvements in the understanding of these issues in recent decades, SJS and TEN continue to create substantial challenges, and unmet needs remain in the management of this disease.

## Author Contributions

TW and JG: conception and design, data collection, writing—original draft review and editing, analysis and interpretation, obtained funding, overall responsibility. CdF, RdA, LM, AdC, FM, and AS'A: data collection, writing—original draft, analysis and interpretation. TB and MdS: conception and design, data collection, writing—original draft, analysis and interpretation. All authors contributed to the article and approved the submitted version.

## Conflict of Interest

The authors declare that the research was conducted in the absence of any commercial or financial relationships that could be construed as a potential conflict of interest.
